# Encephaloid Tumor within the Cranium and on the Neck

**Published:** 1857-11

**Authors:** J. P. Kluge

**Affiliations:** Physician to the Panama Railroad Company


ART. V.—*A Case of Encephaloid Tumor within the Cranium and upon the Front of the Neck.* By J. P. KLUGE, M.D., Physician to the Panama Railroad Company.

CATHERINE LEHMAN, æt. 23, native of Switzerland, was admitted into Ward 24 of the Emigrants' Relief Hospital, New York, on the 30th April, 1853.

*Status Præsens.* She is exceedingly emaciated; pulse ninety-five, small, frequent, and almost threadlike; complexion sallow, or rather of a light straw color; skin dry, harsh, and wrinkled; states she is troubled with profuse and exhausting nightsweats; blepharoptosis and amaurosis affecting left eye; partial paralysis of left inferior extremity. A tumor of the size of a large orange, circumscribed, but immovable, lobulated and firm to the touch, situated over the region of the thyroid gland; at first sight considered to be bronchocele or goitre, as the patient was a native of a section of country where that disease was prevalent and endemic amongst the inhabitants.

Complains of severe cough; sputa, copious, muco-purulent in character, and odor fetid; dyspnœa, probably caused in some degree by the abovementioned tumor compressing the air-passages; subcrepitant râle, commingled with coarse mucous rattling, is heard over the lower portion of right thorax; in the supra-scapular and subclavicular regions of same side expiratory murmur somewhat prolonged, and inspiration indistinct; dulness upon percussion over whole right side of the chest; violent headache, anorexia, and slight mucous diarrhœa. Owing to patient's partially deranged state of mind and great exhaustion, we did not succeed in eliciting anything very satisfactory concerning the previous history of her case, or the circumstances under which the disease occurred. She, however, states to have been sick since last December, and to have had the tumor referred to for three or four years, while still in her native land: for the last two months, she says, it has grown rapidly, notwithstanding the remedial measures employed by a German practitioner, under whose medical charge she had been for some time before applying for admission into the hospital. The fact of the rapid increase of the tumor, and other circumstances, led my colleague, Dr. Walser, and myself, to suspect its being of a malignant character. She further says, that the severity of all her symptoms has been in a corresponding ratio with the sudden enlargement of the tumor, and particularly the pain in her head, which is frontal, and has been unremitting and of an agonizing, lancinating character for the last two weeks.

*Diagnosis.* Not satisfactorily determined; supposed the tumor, however, to be scirrhus or encephaloid cancer, and the patient to be laboring under the cancerous cachexia, with depositions of carcinomatous matter in the brain or its meninges, and perhaps in the parenchyma of the lungs and other viscera.

*Prognosis.* Very unfavorable. *Mors sequetur.*

*Treatment.* Tonics, alteratives with anodynes, to alleviate present symptoms. Syr. Ferri Iodide and Mistura Quiniæ Sulph. in repeated doses during the day; and an anodyne and astringent draught, containing Morph. Sulph. gr. $\frac{1}{4}$, and Plum. Acetas, gr. iii, to be administered at night. Light but nutritious diet. Death took place on the 7th May, after she had been but one week under treatment at the hospital.

*Autopsy.* Made in the presence of the visiting physician, and several members of the resident medical staff, thirty-six hours after death, disclosed the following morbid anatomical appearances. *Brain.* Upon opening the cranium the appearance of the superficies of the brain and medulla oblongata was perfectly normal.

Examination of the base of the brain and cranium disclosed a pulpy, principally dark or bister-colored, sanguineous and encysted tumor, occupying the situation of the pituitary gland in the sella turcica, imbedded in the surrounding substance of the brain. This tumor was of the size of a large walnut: its dimensions were not accurately taken. Having carefully dissected it from the bony structure upon which it principally rested, the sella turcica was discovered to be in a state of caries; posterior clinoid processes entirely obliterated, and anterior ones carious, and in process of absorption.

The external tumor was also removed, and found to resemble lobulated cerebral matter, of a dead-white color in parts, and pinkish and opaque in others, where softening had apparently commenced. In general, however, the mass was rather firm in consistence than otherwise, and upon being cut into, was found interspersed here and there with depositions of a dark brown matter or pigment, tuberculated in appearance, though not according with Abernethy's description of *sarcoma tuberculosum*, although small cavities existed in the vicinity of the tuberculated deposits, which appeared to have resulted from softening or ulceration. The cerebral tumor was in such a state of softening that any structural peculiarities it might have possessed at an earlier period were no longer discernible.

The tumor on the front of the neck had no attachments to the thyroid gland, larynx, or trachea. It was imbedded in the cellular and muscular tissues alone. *Lungs.*—Greatly congested, with œdema of inferior lobes, which pitted upon pressure, and, when incised, exuded a sero-purulent fluid, intermingled with streaks of blood. Superior lobe of right lung in a state of active congestion, with compression and partial obliteration of some of the smaller bronchial tubes. No trace of organic disease could be detected (contrary to expectation) in either the lungs or posterior and anterior mediastinum. Heart normal. Uterus and its appendages healthy, and within the uterus an embryo of probably six weeks or two months was discovered. No morbid appearances discovered in the liver or stomach, with exception of an anemic condition of both. Intestines and remaining viscera not examined, at least I find no mention made in my note-book concerning them.

